# Myocardial Ischemia-Reperfusion and Diabetes: Lessons Learned From Bedside to Bench

**DOI:** 10.3389/fcvm.2021.660698

**Published:** 2021-03-26

**Authors:** Maya Dia, Alexandre Paccalet, Bruno Pillot, Christelle Leon, Michel Ovize, Claire Crola Da Silva, Thomas Bochaton, Melanie Paillard

**Affiliations:** ^1^Laboratoire CarMeN-équipe IRIS, INSERM, INRA, Université Claude Bernard Lyon-1, INSA-Lyon, Univ-Lyon, Bron, France; ^2^Laboratory of Experimental and Clinical Pharmacology, Faculty of Sciences, Lebanese University-Beirut, Beirut, Lebanon; ^3^IHU OPERA, Hospices Civils de Lyon, Bron, France

**Keywords:** diabetes mellitus, myocardia infarction, human, animal model, medication

## Abstract

In front of the failure to translate from bench to bedside cardioprotective drugs against myocardial ischemia-reperfusion, research scientists are currently revising their animal models. Owing to its growing incidence nowadays, type 2 diabetes (T2D) represents one of the main risk factors of co-morbidities in myocardial infarction. However, discrepancies exist between reported animal and human studies. Our aim was here to compare the impact of diabetes on cell death after cardiac ischemia-reperfusion in a human cohort of ST-elevation myocardial infarction (STEMI) patients with a diet-induced mouse model of T2D, using a high-fat high-sucrose diet for 16 weeks (HFHSD). Interestingly, a small fraction (<14%) of patients undergoing a myocardial infarct were diabetic, but treated, and did not show a bigger infarct size when compared to non-diabetic patients. On the contrary, HFHSD mice displayed an increased infarct size after an *in vivo* cardiac ischemia-reperfusion, together with an increased cell death after an *in vitro* hypoxia-reoxygenation on isolated cardiomyocytes. To mimic the diabetic patients' medication profile, 6 weeks of oral gavage with Metformin was performed in the HFHSD mouse group. Metformin treatment of the HFHSD mice led to a similar extent of lower cell death after hypoxia-reoxygenation as in the standard diet group, compared to the HFHSD cardiomyocytes. Altogether, our data highlight that due to their potential protective effect, anti-diabetic medications should be included in pre-clinical study of cardioprotective approaches. Moreover, since diabetic patients represent only a minor fraction of the STEMI patients, diabetic animal models may not be the most suitable translatable model to humans, unlike aging that appears as a common feature of all infarcted patients.

## Introduction

In front of the failure of cardioprotective approaches against myocardial infarction (MI) in several clinical studies (1–3), reconsidering animal models by taking into account all the patients' confounding factors may become inevitable for researchers. Aging, gender, co-morbidities, accompanying diseases and medications are underestimated factors that disrupt the translation of basic research into humans (4). Among them, diabetes appears as one of the most relevant due to its growing rise in prevalence and incidence nowadays.

The World Health Organization reports 422 million people living currently with diabetes worldwide. Diabetes increases the risk of mortality compared with non-diabetic patients and mainly cardiovascular diseases such as stroke and acute coronary syndromes (5, 6). Indeed, diabetes slightly increases the risk of mortality in all type of acute coronary syndromes (7). However, patients with type 2 diabetes (T2D) are often followed and treated with medications to regulate their metabolic dysfunction and these treatments may have an impact on the response to other injury such as an ischemic stress. While a higher risk to develop MI for a diabetic patient is recognized, the effect of diabetes on post-MI infarct size is still not clear. In fact, some studies, focusing on ST-segment elevation myocardial infarction (STEMI), have showed that diabetic patients may develop a larger infarct size, as demonstrated in both clinical trials CORE and EMERALD (8, 9). On the contrary, De Luca et al. have indicated no changes in infarct size between non-diabetic and diabetic patients after primary angioplasty (10). Similar infarct size was also observed in a clinical study comparing diabetic patients with and without insulin treatment (11). Interestingly, these discrepancies among clinical trials mirror the results in diabetic animal studies (12). For example, using the high-fat insulin resistant rat, bigger infarct sizes were reported after *in vivo* cardiac ischemia-reperfusion (13), while the recent study using the T2D Zucker rats showed no difference in infarct size between lean and fatty animals (14).

In the diabetic animal models, the heterogeneity in findings may be related to the diabetic inducers (genetic, treatment, diet…) and/or to the timing (early diabetic cardiomyopathy vs. late heart failure stage); while in patients, the intra- and inter-variabilities between humans, as well as their companion medications schedule could have an impact. It should be noted that *in vivo* cardiac ischemia-reperfusion animal protocols rather represent STEMI patients. In this context, infarct size remains an important determinant of the post-MI outcome and is used as an endpoint in both animal and clinical studies of cardioprotective strategies. Therefore, we ought to investigate further the effect of T2D on myocardial infarct size by confronting the results of STEMI patients to a mouse model of early diabetic cardiomyopathy (15) in order to question the relevance of diabetic animal models in studies of cardioprotection against MI.

## Methods

### Human Cohort and Consent Information

The study was approved by our institution Review Board and Ethics Committee and is registered with the ClinicalTrials.gov identifier NCT03070496. Patients have given their written consent. From the previously described cohort composed of 250 consecutive patients admitted to the Louis Pradel Hospital for a suspected ST-elevation myocardial infarction (STEMI) from 2016 to 2020 (16), all patients underwent coronary angiography at admission with subsequent reperfusion by primary percutaneous intervention (PCI); but only 177 patients underwent contrast enhanced Cardiac Magnetic Resonance (CMR) at one month after MI for infarct size and LV function measurements.

### Type 2 Diabetes Animal Model and *in vivo* Ischemia-Reperfusion Protocol

**Figure 1:**
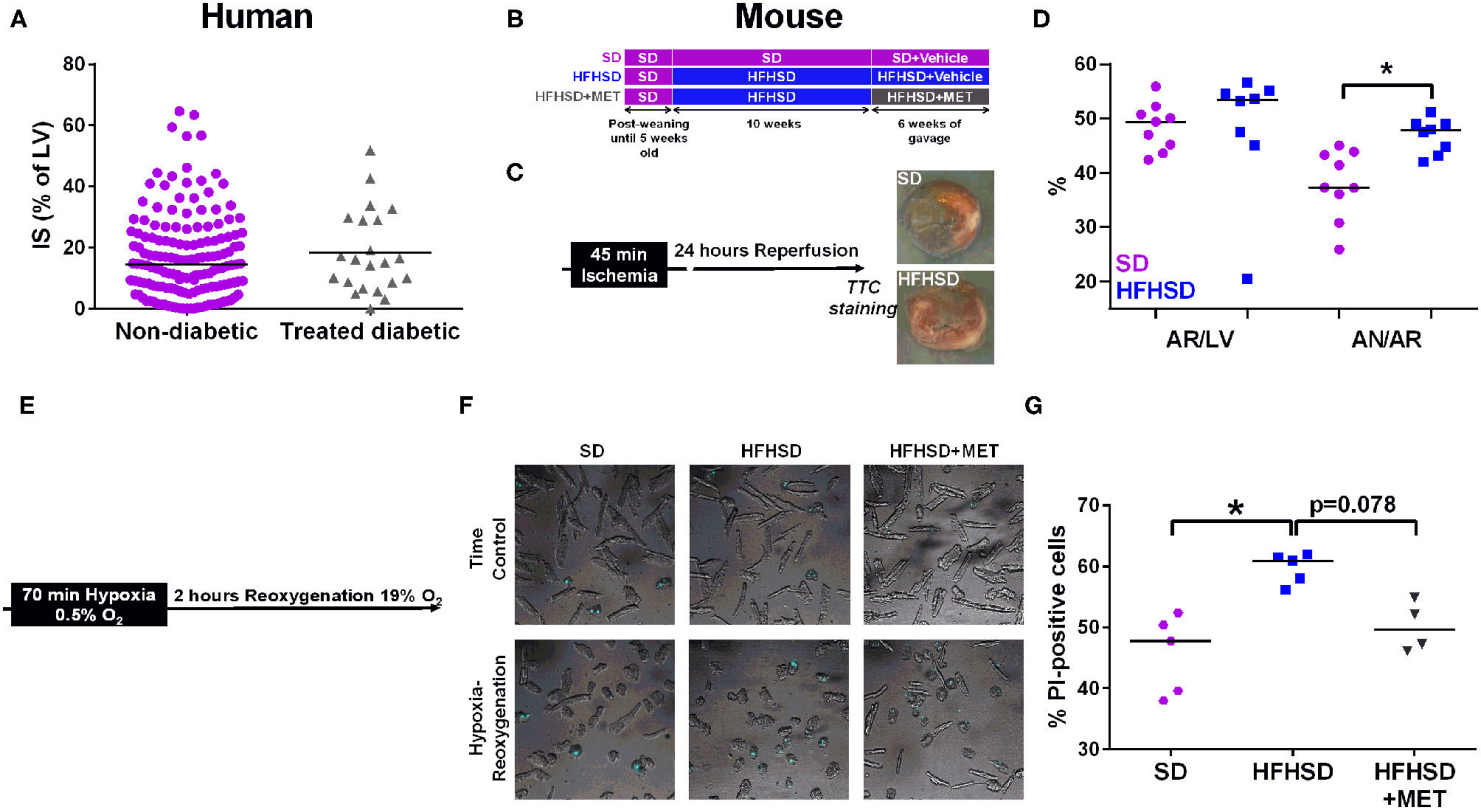
Effect of diabetes on infarct size and cardiomyocyte cell death in humans and mice. **(A)** Measurement of infarct size (IS) as a percentage of the left ventricle (LV), assessed by MRI one month post-MI in patients (155 non-diabetics vs. 22 diabetics). *p* = ns. **(B)** Study design of the diet groups SD and HFHSD together with the oral gavage of Metformin (MET) or Vehicle for the last six weeks of feeding. **(C)** *in vivo* model of myocardial infarction in mice consists in 45 min of ischemia induced by ligation of the left descending coronary artery followed by 24 h of reperfusion before assessment of infarct size. Representative images of infarct size are depicted for each group. **(D)** Measurement of the area at risk (AR/LV), expressed as a percentage of area at risk (AR) over left ventricle (LV), and of the infarct size (AN/AR), calculated as a percentage of area of necrosis over (AN) area at risk. *n* = 9 SD and 8 HFHSD, *p < 0.05. **(E)** Timeline of the hypoxia-reoxygenation protocol: hypoxia is induced for 70 min at 0.5% O_2_ followed by reoxygenation at 19% O_2_ for 2 h before assessment of cell death. **(F)** Representative images of combined white light and propidium-iodide (in blue) cardiomyocytes from SD, HFHSD, and HFHSD+MET mice, in normoxic condition (TC) and after hypoxia-reoxygenation (HR). **(G)** Assessment of cell death by propidium iodide (PI) staining after hypoxia-reoxygenation in cardiomyocytes from SD, HFHSD, and HFHSD+MET mice (*n* = 4-5/group). *p < 0.05.

All animal procedures performed conform to the guidelines from Directive 2010/63/EU of the European Parliament on the protection of animals used for scientific purposes and were approved by the institutional animal research committee from Université Claude Bernard Lyon 1 and the French ministry (#15,627–2018062118508398 and BH2012-65). Male C57BL/6JOlaHsd (Envigo, France) mice were from the same cohort as characterized previously (15): they were subjected to either a standard diet (SD: LASQC diet Rod16-A, Genobios: 16.9% proteins, 4.3% lipids) or a high-fat high-sucrose diet (HFHSD: 260HF U8978 version 19, from SAFE: 20% proteins, 36% lipids, and 35% carbohydrates) for 16 weeks (Figure 1B). For the last 6 weeks of the feeding protocol, metformin gavage (200 mg/kg) was performed daily for half of the HFHSD mice, with the other control group being given the vehicle (0.5% methylcellulose) (17).

For the *in vivo* ischemia-reperfusion (Figure 1C), mice were anesthetized with ketamine (100 mg/kg body weight, intraperitoneal injection) and xylazine (5 mg/kg body weight, intraperitoneal injection) and were orally intubated and ventilated via a rodent ventilator (Physiosuite® system from Kent Scientific). Body temperature was monitored by a rectal thermometer and maintained within the normal range by using a heating pad. A left thoracotomy was performed and the pericardium was opened to expose the heart. An 8-0 polypropylene suture was passed around the left anterior descending artery, under an Euromex microscope, for further creating ischemia. Ischemia was confirmed by ST segment shift on the ECG and appearance of epicardial pallor. After 45 min of coronary artery occlusion, the snare was loosened, and reperfusion was confirmed by visual inspection and reduction of ST segment shift on the ECG. The chest wall was closed with a 5-0 vicryl suture and the endotracheal tube was removed once spontaneous breathing had resumed. The mice were then allowed to recover from the anesthesia in a temperature-controlled area enriched with O_2_. At the end of the 24-h reperfusion, the mice were deeply anesthetized to allow reocclusion of the artery (the suture material was still in place from the previous surgery) and Unisperse blue pigment (0.5 mg/kg i.v.; Ciba-Geigy) was injected in the inferior vena cava. With this technique, the non-ischemic myocardium appears blue, whereas the ischemic myocardium [area at risk (AR)] remains unstained. The heart was then excised and the atrial and right ventricular tissues were removed. The left ventricle was then cut into four 1 mm thick transverse slices. The basal surface of each slice was photographed for later measurement of the area at risk. Slices were then incubated for 15 min in a 1% solution of 2,3,5-triphenyltetrazolium chloride (TTC) at 34°C. With this technique, the viable myocardial tissues appear brick red, whereas the infarcted tissues remain pale. The slices were then photographed for later measurement of the area of necrosis (AN). The extent of the area at risk and the infarcted area was quantified by computerized planimetry and corrected for the weight of the tissue slices.

### Adult Cardiomyocyte Isolation

Mice were heparinized and euthanized by cervical dislocation. Cardiomyocytes were then isolated using O'Connell's protocol (18) and plated for 2 h on glass bottom 35 mm dishes (MatTek) with complete plating medium (MEM Eagle's w/HBSS, FBS 10%, BDM 10 mM, penicillin 100 U/ml, Glutamine 2 mM, ATP 2 mM) prior to the sequence of hypoxia-reoxygenation.

### *In vitro* Hypoxia-Reoxygenation Protocol

Cardiomyocytes were washed twice to remove serum and nutrients with 1 mL Hypoxia Buffer at 37°C (HRB: 0.14 M NaCl, 5 mM KCl, 1 mM MgCl_2_, 10 mM HEPES, 2 mM CaCl_2_, pH 7.4). Hypoxia was performed in 1 mL of HRB solution for 70 min at 0.5% O_2_ / 37°C (including a stabilization period of 25 min to reach the desired level of O_2_ in an hypoxic incubator New Brunswick, Eppendorf). Reoxygenation at 19% O_2_/120 min/37°C was next achieved by quickly but gently replacing the hypoxic medium with 1.5 mL plating medium. At the end of the 2 h of reoxygenation, counting of propidium iodide-positive cells (PI at 1 μg/ml) and of morphologically dead cardiomyocytes (loss of rod-shape) was performed by confocal microscopy (Nikon) and subsequent analysis was done on ImageJ software.

### Statistics

All data were subjected to normality test. Mann-Whitney was applied for the ones which failed the normality test and data were presented as median [Interquartile range]. However, parametric tests were applied for normally distributed data, presented as mean ± SD. For the three groups comparison, a Kruskal-Wallis test followed by a Dunn's multiple comparison test was performed. A *p*-value < 0.05 was considered statistically significant. Analysis was performed on GraphPad Prism.

## Results

### Treated Diabetic Patients Exhibit Similar Infarct Size as the Non-diabetics

Patients were included with a median age of 59 ± 12 years. Among the 250 patients, 37 patients suffered from type 2 diabetes, giving a proportion of 14.8% diabetic patients suffering from MI in this cohort. The characteristics of the study population are presented in Table 1. Type 2 diabetic patients were significantly older than non-diabetic patients with a median age of 62 ± 11 vs. 57 ± 12 years (*p* < 0.05), and were more prone to hypertension (Table 1). Diabetic patients had a higher percentage of medications taken, including aspirin, statins, betablockers, and angiotensin-converting enzyme inhibitors/angiotensin II receptor blockers (ACEi/ARB) (Table 1). All diabetic patients were treated with antidiabetics, mainly Metformin and 9 were under insulin, therefore being considered as treated diabetic. Based on the analysis of the Killip Status, we observed a higher rate of patients with sign of heart failure development in the treated-diabetic patients group compared to the non-diabetic patients (Table 1). No differences were observed between the two population regarding the coronary flux evaluated with the TIMI flow grade. Treated-diabetic patients displayed a higher level of C-reactive protein compared to non-diabetic patients (42.6 mg/L interquartile range (IQR): [11.7-74.3] compare to 16.1 mg/L IQR [7.1-40.5], *p* = 0.02). In our cohort population, dyslipidemia affected more patients with treated diabetes than non-diabetic patients (23.5% compare to 62.1%, *p* < 0.001).

**Table 1:** Characteristics of the study population.

	**Non-diabetic patients (*n* = 213)**	**Diabetic patients (*n* = 37)**	***p* value**
Age, years	57 ± 11	63 ± 10	0.02
Male sex, nb (%)	168 (78.9)	30 (81.1)	0.83
Body Mass Index (BMI), kg/m^2^	26.3 [23.9-29.4]	25.9 [23.7-29.6]	0.69
Dyslipidemia, nb (%)	50 (23.5)	23 (62.1)	<0.0001
Hypertension, nb (%)	52 (24.4)	22 (59.5)	<0.0001
Current smoker, nb (%)	150 (70.4)	21 (56.8)	0.12
Time from symptoms to PCI, min	205 [145-334.0]	200.0[120.0-251.3]	0.46
Anterior MI, nb (%)	113 (53.3)	19 (51.4)	0.86
Killip status = 1, nb (%)	193 (90.5)	31 (79.5)	0.05
TIMI flow grade at admission = 0-1, nb (%)	140 (72.5)	28 (75.7)	0.84
Post-PCI TIMI flow grade >2 (%)	207 (98.1)	34 (91.9)	0.07
LVEF at baseline (%)	55.0 [46.0-61.3]	50.0 [44.0-62.0]	0.2957
Peak troponin I, ng/L	43907 [16642-107843]	46466 [14353-144943]	0.55
Peak creatine kinase, mUI/L	1529 [684.3-3542.0]	1815 [641.0-4076.0]	0.68
Peak CRP, mg/L	16.1 [7.1-40.5]	42.6 [11.7-74.3]	0.02
Aspirin, nb (%)	24 (11.3)	10 (27.0)	0.02
Betablockers, nb (%)	17 (8.0)	9 (24.3)	<0.0001
ACEi / ARB, nb (%)	27 (12.7)	17 (45.9)	0.006
Statins, nb (%)	21 (9.9)	13 (35.1)	0.0002

*Values are expressed as Mean ± SD, Median [interquartile range] or number with percentage (%). PCI, percutaneous coronary intervention; MI, myocardial infarction; TIMI, thrombolysis in myocardial infarction; LVEF, left ventricular ejection fraction; CRP, C-reactive protein. ACEi, angiotensin-converting enzyme inhibitors; ARB, angiotensin II receptor blockers*.

No differences were observed neither on the infarct size measurement between treated-diabetic and non-diabetic patients (Figure 1A) (respectively, 14.5% [6.8-24.2] of the left ventricle mass compared to 15.6% [IQR: 8.1-29.1]), nor on the left ventricular ejection fraction assessed at one month (treated-diabetic, 53% [IQR: 46.0-58.5] compared to non-diabetic, 50.5 [IQR: 41.5-57.0]).

### Increased Cell Death After Hypoxia-Reoxygenation in Diabetic Cardiomyocytes Is Prevented by Metformin Treatment

The mice used in this study were from the same cohort in which we previously characterized the diet-induced T2D mouse model recapitulating the early stage of diabetic cardiomyopathy in human, notably glucose, and insulin intolerance, hyperglycemia, and hyperlipidemia (15). We thus investigated the effect of T2D on infarct size after an *in vivo* ischemia-reperfusion sequence in the 16 weeks diet-fed mice, at the age of 21 weeks (Figure 1C). While the areas at risk were comparable between SD and HFHSD mice, HFHSD displayed a significant bigger infarct size compared to the SD mice (Figure 1D: HFHSD, 47.8 [43.5, 49.0] vs. SD, 37.3 [33.5, 43.6] % AR/AN, *n* = 8-9 mice/group, *p* < 0.05). Similarly, cardiomyocytes freshly isolated from HFHSD diabetic mice displayed a significant increased cell death upon simulated ischemia-reperfusion, namely hypoxia-reoxygenation, compared to the SD cardiac cells (Figures 1E–G, HFHSD, 60.9 [57.1, 61.8] vs. SD, 47.8 [38.8, 51.4] % of PI-positive cells, *n* = 5/group, *p* < 0.05).

One could wonder whether the difference in diabetes-induced cell death after an ischemic event between the patients and the mouse model could rely on the antidiabetic medication regimen taken by the diabetic patients of the cohort, notably Metformin. To this end, the HFHSD mice received an oral gavage with Metformin for the last 6 weeks of the feeding. As previously described, Metformin did not decrease the body weight of the HFHSD mice but partially rescued the sensitivity to insulin and glucose (17) (data not shown). Interestingly, Metformin treatment of the HFHSD mice led to a reduction of cell death after hypoxia-reoxygenation compared to the HFHSD cardiomyocytes (HFHSD+MET, 49.7 [46.4, 54.2] % of PI-positive cells, *n* = 4-5/group, *p* = 0.078 vs. HFHSD), reaching a similar extent as in the standard diet group (*p* = ns vs. SD) (Figures 1F,G).

## Discussion

The goal of our study was to compare the impact of diabetes on cell death after cardiac ischemia-reperfusion in a human cohort of STEMI patients with a diet-induced mouse model of T2D. In our human study population, no differences have been observed regarding the infarct size between treated-diabetic and non-diabetic patients. The baseline characteristics of our study populations are in line with the literature. Indeed, dyslipidemia is a common feature of diabetes (19) and diabetic patients displayed a higher level of C-reactive protein (20). Our population cohort displayed a small proportion of diabetic patients (14.8 %), which may be explained by the selection of only STEMI patients. Indeed, the proportion of diabetic patients in non-STEMI population is more important than in STEMI, as previously reported in the FAST-MI study (21) (16.5% of STEMI patients are diabetic while 27% of non-STEMI patients are diabetic) and in a larger database study (7) (28.8% of non-STEMI patients were diabetic compared to 18.2% in the STEMI group). Interestingly, a temporal study between 1995 and 2003 also highlighted that the diabetic patients are now more prompt to non-STEMI events (5).

As to our results in the HFHSD mice, they revealed an increased infarct size in an *in vivo* model of myocardial infarction as well as an increased cell death following a simulated ischemia-reperfusion, which was prevented by a Metformin daily treatment, as usually performed in diabetic patients. Importantly, Metformin has been shown to exert a cardioprotection through activating the AMPK pathway and upregulating PGC-1α, which improves mitochondrial organization and function (22, 23). Metformin is used as a first line antidiabetic drug, not only due to its glucose lowering potential, but also to its cardiovascular safety and protective contribution. Altogether, our data suggest that the similar infarct size seen between all STEMI patients regardless of their diabetic history may be mainly due to the protective effect afforded by their antidiabetic medication, such as Metformin, insulin, and sulfonylurea drugs, as previously described (13, 24). Therefore, evaluation of the protective effect of new therapeutic drugs in diabetic animal models is effectively of interest but should be combined with the routinely used antidiabetic medications, such as Metformin, to rule out any confounding action between the pre-existing medications and the potential protective therapy. However, while similar results were observed after *in vivo* and *in vitro* ischemia-reperfusion, i.e., increased infarct size/cell death in the HFHSD group vs. SD group, thus validating the relevance of the *in vitro* experiments, one limitation of our study relies on the absence of *in vivo* measurement of infarct size in the HFHSD+MET group. Moreover, besides their antidiabetic treatments, diabetic patients were significantly receiving more treatments than non-diabetic patients, i.e., aspirin, betablockers, and ACEi, which may also have cardioprotective effects not directly on infarct size but on the major adverse cardiovascular events (25). Therefore, taking into consideration also the current medications used routinely by the patients and at the time of reperfusion in the clinical settings would be an invaluable asset in assessing the relevance of cardioprotective drugs in *in vivo* animal studies.

However, as shown by our human cohort of STEMI patients and by others, diabetic patients only represent a minor fraction (around 15%) of the STEMI patients while closer to 30% in the non-STEMI population. Since the current animal models of *in vivo* ischemia-reperfusion usually rely on a coronary artery occlusion, thus representing the STEMI population, diabetes may not be the best co-morbidities to be taken into account to evaluate new cardioprotective strategies. While aging and hypertension were significantly more present in the diabetic patients and could also explain a form of cardioprotection by favoring coronary collateral circulation (26), one main common factor of all patients is aging (4). Although this factor is more complicated to pursue in animal models, future studies are required to assess the relevance of aging in our animal models to study cardioprotection against MI.

Finally, it has been recently shown that infarct size is not the only factor to take into consideration regarding the patient post-MI clinical outcome (25), therefore highlighting the importance to evaluate the effect of cardioprotective strategies not only on infarct size but on the contractile function and the survival notably. Indeed, the higher rate of mortality observed in diabetic patients after MI may not be due to the infarct size but to several confounding factors observed in diabetic patients such as the diabetic cardiomyopathy and dyslipidemia.

All these discrepancies between studies, whether among animal models or clinical testing, have raised a lot of questioning about scientists' attempt to translate their fundamental research into clinics. Here, we provide some advices for translational research in the field of cardioprotective strategies against MI by: (1) considering comorbidities, such as diabetes, together with their daily medication, into the animal models; (2) evaluating the relevance of each comorbidity in the protective approaches, notably between diabetes and aging; and (3) extending our experimental endpoints beyond infarct size, i.e., contractile function and survival study.

## Data Availability Statement

The raw data supporting the conclusions of this article will be made available by the authors, without undue reservation.

## Ethics Statement

The studies involving human participants were reviewed and approved by CPP of the Hospices Civils de Lyon. The patients/participants provided their written informed consent to participate in this study. The animal study was reviewed and approved by Université Claude Bernard Lyon 1 and the French ministry.

## Author Contributions

MP, MD, AP, CC, and TB: conceptualization. MD, CL, BP, and MP: experimental investigation. MD, AP, TB, and MP: data collection and analysis. MD and AP: manuscript writing. MP, TB, and CC: manuscript reviewing. MP and MO: funding acquisition. All authors contributed to the article and approved the submitted version.

## Conflict of Interest

The authors declare that the research was conducted in the absence of any commercial or financial relationships that could be construed as a potential conflict of interest.
